# Anchorage-Capture Dual Mechanism in a Biomass-Derived Hydrogel Electrolyte for Dendrite-Free Aqueous Zinc Ion Batteries

**DOI:** 10.3390/polym18161957

**Published:** 2026-08-10

**Authors:** Shubing Zhen, Yali Song, Jingyu Xu, Xinhao Li, Jiayuan Luo, Yuyun Xie, Jinxi Ye, Yushi Wu, Guiling Wang, Qian Qu, Tong Zhang

**Affiliations:** 1 School of Mechanical and Electrical Engineering, Guangdong University of Science and Technology, Dongguan 523086, China; zhenshubing1@gdust.edu.cn (S.Z.); xujingyu5@gdust.edu.cn (J.X.); lixinhao@gdust.edu.cn (X.L.); luojiayuan@gdust.edu.cn (J.L.); xieyuyun@gdust.edu.cn (Y.X.); yejinxi@gdust.edu.cn (J.Y.); wuyushi@gdust.edu.cn (Y.W.); 2 Key Laboratory of Superlight Materials and Surface Technology, Ministry of Education, College of Materials Science and Chemical Engineering, Harbin Engineering University, Harbin 150001, China; songyali@hrbeu.edu.cn (Y.S.); wangguiling@hrbeu.edu.cn (G.W.)

**Keywords:** biopolymer electrolyte, dual-network hydrogel, zinc anode protection

## Abstract

The design of biomass-derived polymer electrolytes with integrated multifunctionality represents a key strategy for sustainable energy storage devices. Here, we report a fully biomass-derived dual-network hydrogel electrolyte fabricated by combining Pectin (PC) and Chitosan (CTS), two naturally abundant polysaccharides. The Pectin/Chitosan dual-network hydrogel electrolyte (PC/CTS) forms a robust physically crosslinked network through electrostatic interactions between the carboxyl groups of PC and the amino groups of CTS, reinforced by dense hydrogen bonding and amide crosslinks, yielding a tensile strength of 77.76 MPa. The abundant polar functional groups of the dual polymer network serve a synergistic dual function: the amino groups of CTS preferentially adsorb onto the zinc anode surface (adsorption energy: −1.24 eV), forming a dynamic protective interphase, while the carboxyl groups of PC coordinate with Zn^2+^ (binding energy: −0.86 eV), reconstituting the solvation sheath and guiding uniform ion flux. This anchorage-capture mechanism, enabled by the molecular design of the polymer network, effectively suppresses dendrite growth, hydrogen evolution, and parasitic side reactions. Consequently, the PC/CTS electrolyte enables stable Zn//Zn cycling for 3350 h, 99.5% average Coulombic efficiency (CE) over 780 Zn//Cu cycles (at 5 mA cm^−2^ and 1 mAh cm^−2^), and 63.8% capacity retention after 500 cycles in Zn//MnO_2_ full cells. This work demonstrates that rational engineering of natural polymer networks can simultaneously address electrode stability challenges in aqueous batteries, offering a sustainable materials platform for next-generation energy storage devices.

## 1. Introduction

Hydrogel electrolytes have emerged as a promising polymer materials platform for aqueous energy storage devices, combining high ionic conductivity with mechanical flexibility and device safety [1,2]. However, their practical application is limited by interfacial instabilities at the electrode surface, where uncontrolled dendrite growth, hydrogen evolution reaction (HER), and progressive corrosion degrade performance and shorten lifetime [3,4]. To address these challenges, various polymer-based strategies have been developed, including electrolyte additives, artificial protective layers, and solid-state electrolyte designs [5,6]. Among these, single-network biopolymer hydrogels have been widely studied for their straightforward fabrication and biocompatibility [7]. Yet these single-network systems can typically address only one aspect of the interfacial instability problem at a time, in that a hydrogel designed for mechanical integrity may not regulate ion solvation, while one designed for interfacial passivation may lack sufficient mechanical strength (typically <25 MPa) [8].

A different polymer design is therefore needed, one that can simultaneously provide mechanical robustness, active regulation of ion solvation, and dynamic interfacial protection within a single materials platform. Natural polysaccharides offer an attractive chemical toolkit for this purpose, with their abundant and diverse polar functional groups (carboxyl, amino, hydroxyl) capable of multiple modes of interaction with both ions and electrode surfaces [9,10]. When designed as dual-network architectures, complementary biopolymers can achieve mechanical properties and chemical functionalities that neither polymer alone can provide [11,12]. Although hydrogel electrolytes with reduced water content have been reported and can effectively mitigate water-induced side reactions, such strategies mainly rely on the passive physical regulation of water, whereas polysaccharide chains can provide active chemical regulation of both the solvation sheath and the electrode interface through their abundant polar functional groups [13,14].

Here we demonstrate that this dual regulation is achievable in PC/CTS electrolyte. The carboxyl groups of PC preferentially coordinate with Zn^2+^ ions (binding energy: −0.86 eV), displacing water molecules from the solvation sheath to lower the desolvation energy barrier, while the amino groups of CTS preferentially adsorb onto the zinc electrode surface (adsorption energy: −1.24 eV), forming a dynamic protective interphase [13,14]. We term this dual action the anchorage-capture mechanism. In contrast to strategies based mainly on physical water-content control, this mechanism originates from the intrinsic chemical functionality of the polymer chains, enabling simultaneous interfacial protection and solvation-structure regulation. The resulting electrolyte achieves a tensile strength of 77.76 MPa, stable Zn//Zn cycling for over 3350 h, 99.5% average CE over 780 cycles, and 63.8% capacity retention in Zn//MnO_2_ full cells after 500 cycles. This work establishes that molecular engineering of naturally occurring polysaccharide pairs can create advanced polymer electrolytes capable of simultaneously addressing bulk transport and interfacial stability challenges in energy storage devices. Furthermore, PC and CTS are abundant biomass-derived polysaccharides that can be processed in water without organic synthesis or purification, offering potential advantages in cost and scalable manufacturing. The biodegradable polymer network further provides an environmentally favorable electrolyte component for sustainable energy storage.

## 2. Results and Discussion

### 2.1. Design and Physicochemical Characterization of the Dual-Network Hydrogel

**Figure 1:**
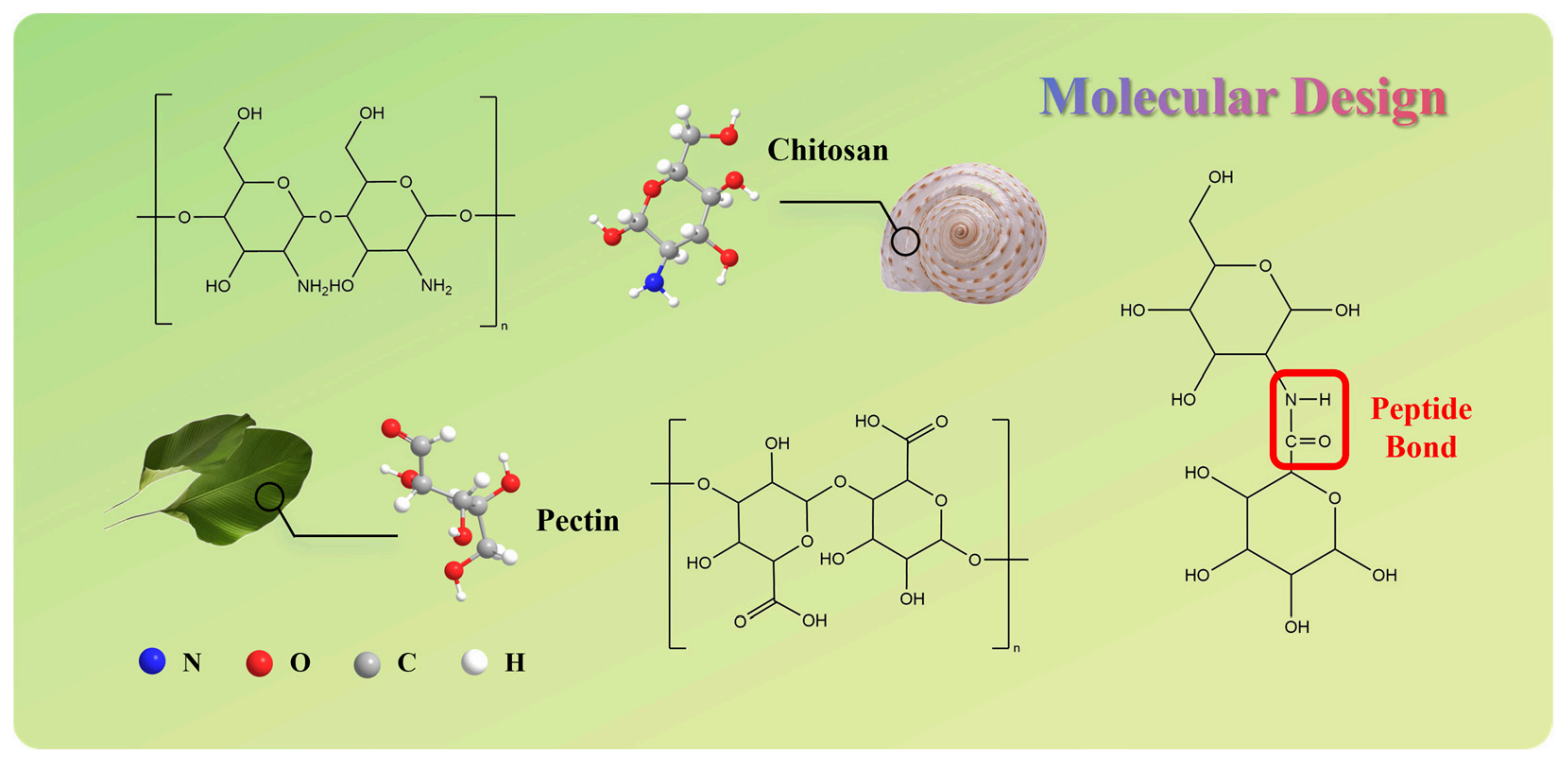
Schematic illustration of the structures of PC and CTS.

To achieve a stable zinc anode interface, we designed a crosslinked dual-network hydrogel electrolyte leveraging electrostatic complexation between CTS and PC, as illustrated in Figure 1. Under acidic preparation conditions, the positively charged amino groups (-NH_2_) on CTS chains and the negatively charged carboxyl groups (-COOH) on PC chains undergo spontaneous electrostatic complexation, forming the primary physical crosslinking network. This is further reinforced by dense intermolecular hydrogen bonding among the abundant -OH, -COOH, and -NH_2_ functional groups, establishing a robust three-dimensional network structure [15,16]. To verify the acidity of the preparation system, the pH of the PC/CTS mixture before gelation was measured at approximately 3.45, which is within the mildly acidic range that preserves PC stability and promotes electrostatic complexation. After equilibration in 2 M ZnSO_4_, the hydrogel electrolyte exhibited a pH of 3.41, slightly higher than that of the pristine 2 M ZnSO_4_ solution (2.29). This slight increase is attributed to the buffering effect of the PC/CTS polymer network, which moderates the acidity of the electrolyte and is expected to contribute to suppressing hydrogen evolution during battery operation.

**Figure 2:**
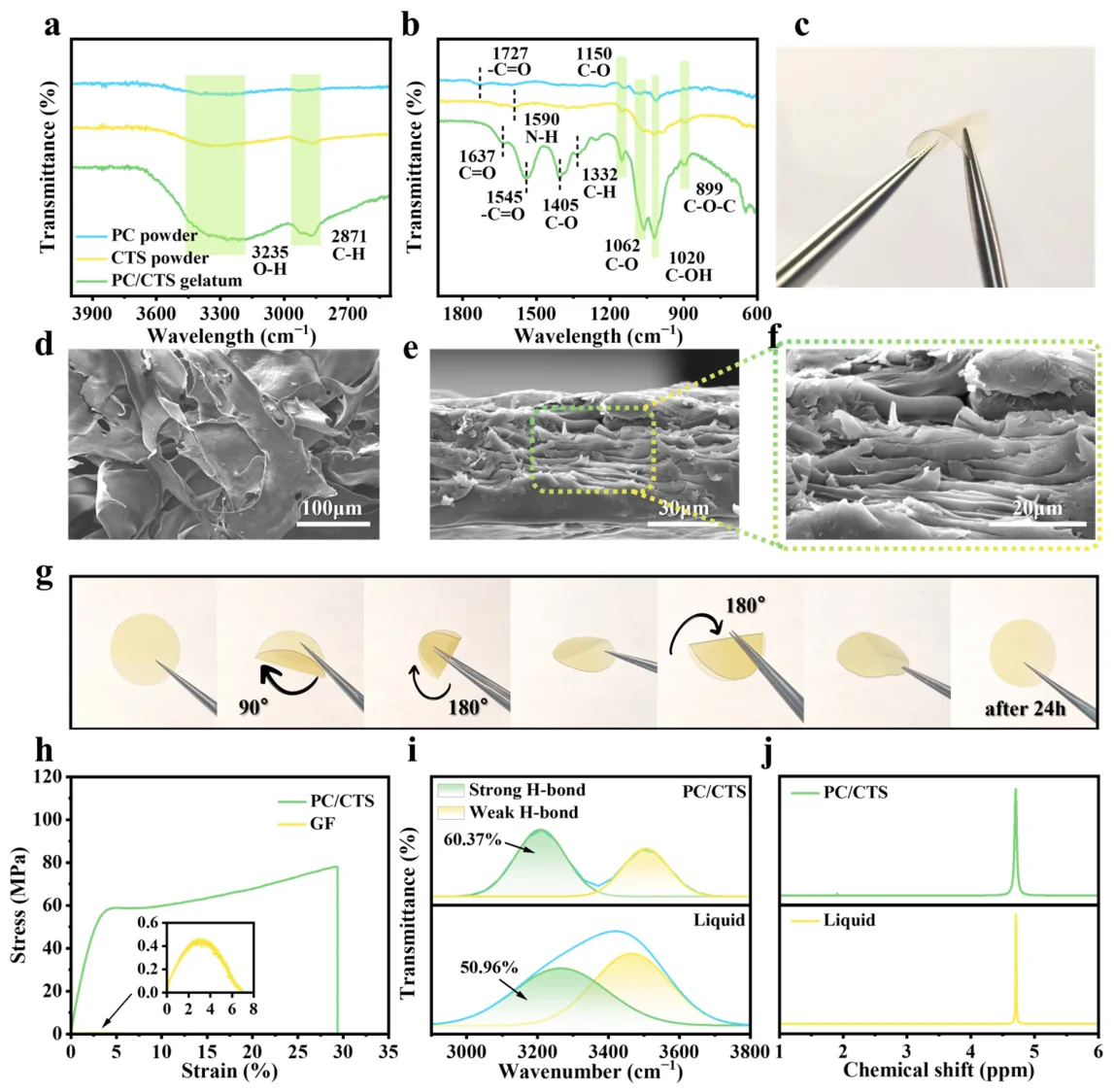
(**a**,**b**) FTIR spectra of PC, CTS, and PC/CTS membrane; (**c**) Photograph of the PC/CTS; (**d**) SEM image of the PC/CTS membrane; (**e**,**f**) Cross-sectional SEM images of the PC/CTS membrane; (**g**) Photographs of the folding process of PC/CTS membrane; (**h**) Tensile stress-strain curves of PC/CTS and GF separator; (**i**) Raman spectra of PC/CTS electrolyte and liquid ZnSO_4_ electrolyte; (**j**) 1H nuclear magnetic resonance (NMR) spectra of PC/CTS electrolyte and liquid electrolyte.

Fourier transform infrared spectroscopy (FTIR) confirmed the chemical crosslinking reaction between PC and CTS (Figure 2a,b). All samples exhibit a broad and intense absorption band at 3000–3600 cm^−1^, corresponding to -OH stretching vibrations from the abundant hydroxyl functionalities on the PC and CTS molecular chains [17,18]. Notably, the characteristic -NH_2_ bending peak of pristine CTS at 1590 cm^−1^ completely disappears in the PC/CTS, while two new absorption peaks emerge at 1637 cm^−1^ (amide I band, -C=O stretching) and 1545 cm^−1^ (amide II band, N-H bending). These newly appeared peaks are consistent with the formation of amide crosslinks (-CO-NH-) during the drying step [11,12], in addition to electrostatic complexation and hydrogen bonding, contributing to a stable multi-network structure that provides the structural foundation for the superior mechanical properties of the material.

The PC/CTS exhibits a translucent light-yellow appearance with good macroscopic uniformity (Figure 2c). Scanning electron microscopy (SEM) imaging (Figure 2d–f) reveals a well-ordered lamellar microstructure with organized layered features, which provides directional transport channels for Zn^2+^ and guides the uniform distribution of ions at the electrode surface, thereby promoting homogeneous zinc nucleation and deposition [19]. The hydrogel thickness is approximately 53 um (Figure S1), substantially thinner than a conventional glass fiber (GF) separator (194 um), which significantly shortens the Zn^2+^ transport pathway between cathode and anode, enhancing ionic conduction efficiency and improving the volumetric energy density of the battery [20,21]. Excellent mechanical properties are critical for the practical application of the hydrogel electrolyte, particularly for flexible energy storage devices. The PC/CTS rapidly recovers its original shape after repeated ±180 bending without any structural damage, demonstrating its outstanding flexibility (Figure 2g). Quantitative tensile testing (Figure 2h) shows a high tensile strength of 77.76 MPa and an elongation at break of 29.36%, which are substantially superior to those of a conventional GF separator (tensile strength ~0.45 MPa and elongation at break ~3.2%). This outstanding mechanical performance derives from the cooperative reinforcement of the dual-network structure, combining electrostatic interactions, hydrogen bonding, and amide crosslinks, providing a critical material foundation for integration into flexible energy storage devices [22,23].

We further investigated the state of water molecules and the hydrogen-bonding network within the PC/CTS after immersion in zinc sulfate electrolyte. Raman spectroscopy of the O-H stretching vibration region (Figure 2i) shows that the proportion of strong hydrogen bonds in the PC/CTS electrolyte reaches 60.37%, significantly higher than that in liquid electrolyte (50.96%), confirming that the abundant polar functional groups on PC and CTS chains not only form dense intermolecular hydrogen bonds with each other but also reconfigure and strengthen the hydrogen bonding network of the electrolyte system [24]. Consistent with this, 1H NMR spectroscopy (Figure 2j) shows that the characteristic water peak at approximately 4.7 ppm is significantly broadened in the PC/CTS electrolyte compared to the liquid electrolyte, indicating that water mobility is substantially restricted within the three-dimensional PC/CTS network [25,26]. This dual confinement mechanism, physical entrapment by the dense polymer network and chemical anchoring through strong hydrogen bonding interactions, fundamentally reduces the activity of free water. As a result, the PC/CTS exhibits excellent water retention capability compared to conventional GF separators (Figure S2), providing bulk-phase assurance for suppressing HER and interfacial side reactions at the zinc anode [27,28,29].

### 2.2. Anchorage-Capture Mechanism: Density Functional Theory (DFT) and Mechanistic Insights

**Figure 3:**
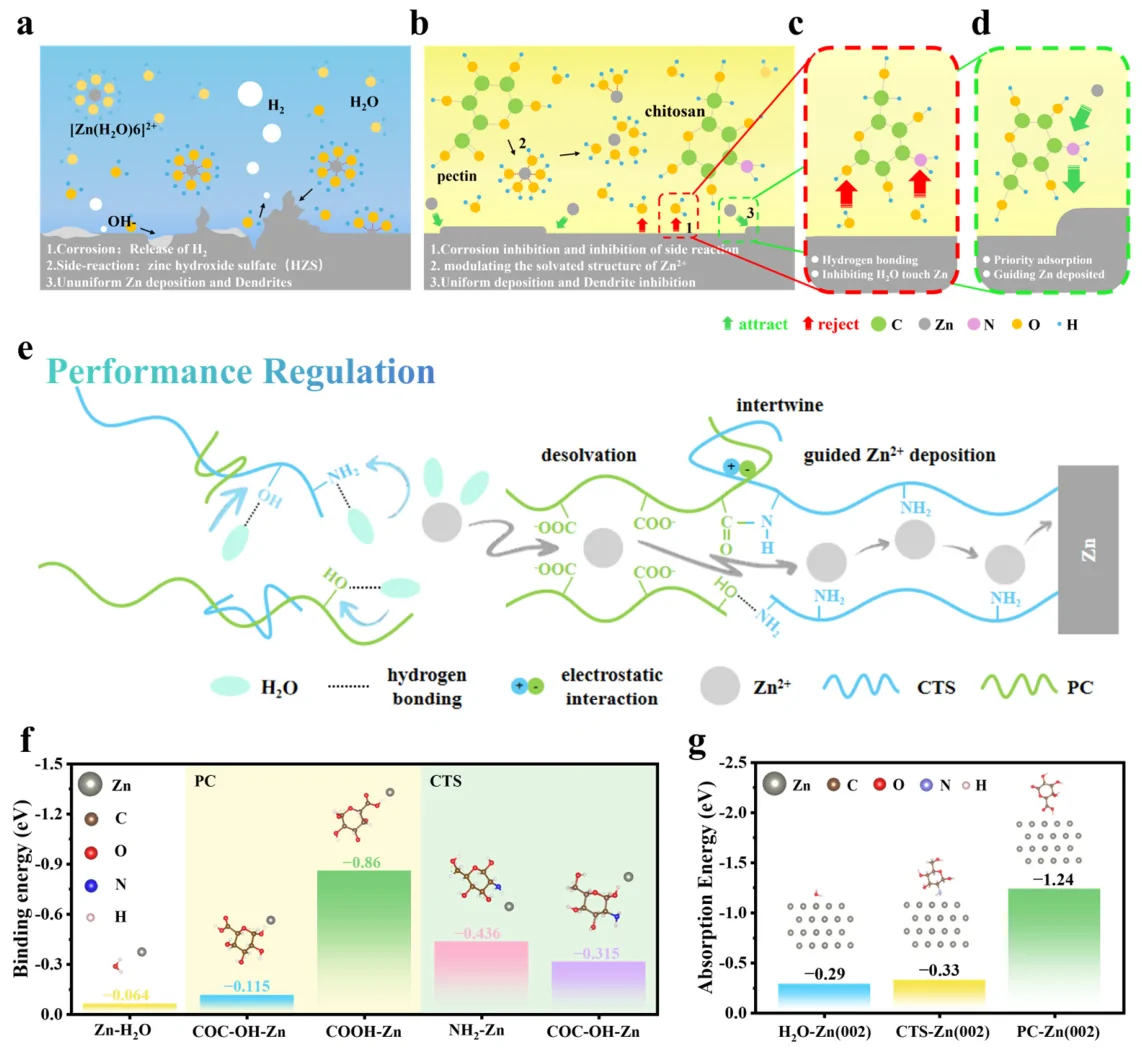
(**a**) Schematic illustration of the Zn^2+^ deposition process in liquid electrolyte; (**b**–**d**) Schematic illustrations of the Zn^2+^ deposition process in the PC/CTS electrolyte; (**e**) Schematic diagram of the synergistic mechanism for Zn^2+^ deposition regulation in the PC/CTS electrolyte; (**f**) Binding energy diagrams of H_2_O, PC, and CTS with Zn^2+^; (**g**) Adsorption energy diagrams of H_2_O, PC, and CTS on the Zn (002) crystal plane.

Figure 3a–e illustrate the key physicochemical processes at the zinc anode/electrolyte interface in a conventional liquid electrolyte versus the PC/CTS. In the conventional aqueous electrolyte (Figure 3a), active water molecules come into direct contact with the zinc metal, triggering continuous HER and corrosion [30]. HER consumes H^+^ at the interface, leading to a significant increase in local pH, which promotes the irreversible formation of Zn_4_SO_4_(OH)_6_·xH_2_O byproducts. These byproducts passivate the electrode surface, exacerbate the inhomogeneous distribution of the interfacial electric field and Zn^2+^ flux, and ultimately result in localized zinc accumulation and rampant dendrite growth. In contrast, the PC/CTS dual-network hydrogel achieves interfacial stabilization through a synergistic dual mechanism (Figure 3b–e). The positively charged amino groups on the CTS chains preferentially adsorb onto the zinc anode surface, forming a dynamic protective layer that blocks direct water contact and suppresses HER and corrosion [31]. Meanwhile, the negatively charged carboxyl groups on the PC chains exhibit high affinity for Zn^2+^, partially replacing water molecules in the primary solvation sheath, reconstituting the solvation structure, lowering the desolvation energy barrier, and guiding Zn^2+^ migration along the polymer chains [32].

To explore this synergistic anchorage-capture mechanism at the molecular level, we performed systematic DFT calculations. Electrostatic potential analysis (Figure S3) reveals complementary charge distributions: PC molecules exhibit a distinct negative electrostatic potential region at the carboxyl sites, whereas CTS molecules display positive electrostatic potential around the amino groups, providing the electrostatic basis for stable dual-network formation through electrostatic interactions. Building on this, we further evaluated the binding energies between different components and Zn^2+^. The calculated binding energies of Zn^2+^ with PC carboxyl groups (−0.86 eV) and CTS amino groups (−0.44 eV) are both significantly higher than that between water and Zn^2+^ (−0.064 eV) (Figure 3f), confirming that Zn^2+^ preferentially coordinates with PC and CTS molecules [33]. Such preferential coordination effectively reconstitutes the primary solvation sheath of Zn^2+^, displacing active water molecules from its coordination environment and substantially reducing the desolvation energy barrier at the electrode interface. Adsorption energy calculations on the Zn(002) plane (Figure 3g) show that CTS exhibits the strongest adsorption (−1.24 eV), followed by PC (−0.33 eV), both significantly stronger than that of water (−0.29 eV). Notably, the exceptionally strong adsorption of CTS indicates it can spontaneously and preferentially adsorb onto the zinc electrode surface, forming an effective interfacial protective layer [34]. Collectively, the DFT calculations elucidate the synergistic stabilization mechanism from two interconnected perspectives: bulk-phase solvation-structure reconstruction via competitive coordination, and interface-level composite protective layer formation through co-adsorption of PC and CTS. Together, these effects ensure high reversibility of the zinc deposition process.

### 2.3. Electrochemical Performance and Interfacial Stability

**Figure 4:**
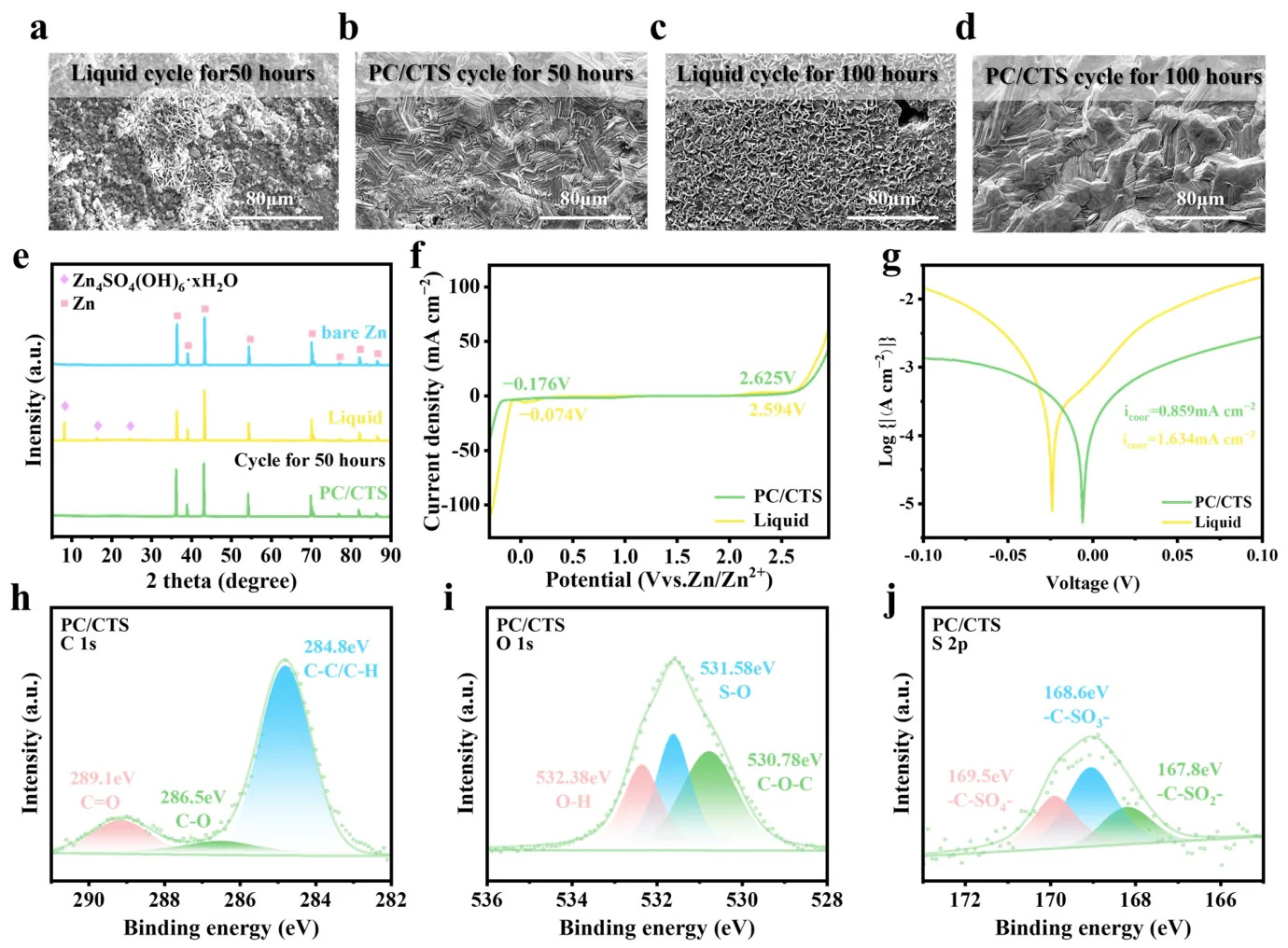
SEM images of Zn anodes cycled in (**a**) liquid electrolyte and (**b**) PC/CTS electrolyte for 50 h; corresponding SEM images after 100 h of cycling in (**c**) liquid electrolyte and (**d**) PC/CTS electrolyte. (**e**) XRD pattern of the Zn anode after 100 cycles at 1 mA cm^−2^ and 1 mAh cm^−2^. (**f**) Linear sweep voltammetry (LSV) curves and (**g**) Tafel polarization curves. (**h**–**j**) X-ray photoelectron spectroscopy (XPS) spectra of the Zn anode cycled in PC/CTS electrolyte at 1 mA cm^−2^ and 1 mAh cm^−2^ for 100 h: (**h**) O 1s, (**i**) C 1s, and (**j**) S 2p.

To systematically evaluate the regulatory effect of the PC/CTS electrolyte on zinc deposition behavior, we conducted comprehensive electrochemical tests coupled with ex situ characterization. SEM imaging of the zinc anode before (Figure S4) and after cycling provides direct morphological evidence (Figure 4a–d and Figure S5). The pristine surface is relatively smooth and only exhibits polishing scratches; after cycling for 50 h in ZnSO_4_ liquid electrolyte, the zinc surface is severely roughened with abundant needle-like dendrites and byproduct accumulation, while the PC/CTS electrolyte maintains a smooth, dense, dendrite-free surface. After 100 h, the contrast becomes even more pronounced: the liquid electrolyte system shows catastrophic degradation, whereas the PC/CTS system retains a compact morphology with flat growth features. In the liquid electrolyte, the severe morphological degradation originates from the tip effect: Zn^2+^ preferentially deposits at microscopic protrusions, and the positive feedback between localized ion accumulation and insufficient deposition elsewhere promotes dendrite growth, which in turn accelerates hydrogen evolution, corrosion, and dead-zinc formation. The PC/CTS hydrogel suppresses these coupled processes through the anchorage-capture mechanism and maintains a dense, dendrite-free surface. X-ray diffraction (XRD) analysis (Figure 4e) confirms the absence of Zn_4_SO_4_(OH)_6_·xH_2_O diffraction peaks in the PC/CTS system after cycling, demonstrating effective suppression of parasitic side reactions [35,36].

We further evaluated the interfacial stability of the zinc anode using electrochemical techniques. LSV results (Figure 4f) show that the PC/CTS electrolyte exhibits a wider electrochemical stability window compared to the liquid electrolyte, with the HER onset potential shifted negatively by approximately 102 mV and the oxidation potential shifted positively by about 31 mV, indicating effective broadening of the operating voltage range and enhanced interfacial stability. Tafel analysis (Figure 4g) reveals that the zinc electrode in the PC/CTS electrolyte exhibits a significantly positive shift in corrosion potential, while the corrosion current density is substantially reduced from 1.064 to 0.858 mA cm^−2^, confirming effective suppression of zinc corrosion [37,38]. XPS analysis after the 100 h cycle (Figure 4h,i and Figure S6–S9) provides direct chemical evidence for the formation of a dynamic organic protective layer on the zinc surface. In the C 1s spectra (Figure 4h and Figure S6), a prominent C=O characteristic peak appears at 288.9 eV for the PC/CTS group with substantially higher intensity than in the liquid electrolyte group, indicating enrichment of organic species on the zinc anode surface. The O 1s spectra (Figure 4i and Figure S7) exhibit a significantly enhanced -OH peak at 532.38 eV and the appearance of a C-O-C feature at 530.78 eV, attributed to organic moieties from PC and CTS that adsorb and accumulate during cycling [39]. The S 2p spectra (Figure 4j and Figure S8) show markedly diminished sulfate (SO_4_^2−^) peak intensity in the PC/CTS group, confirming effective suppression of sulfate-related side reactions, consistent with the XRD results [40,41]. In the Zn 2p spectra (Figure S9), the binding energy shifts from 1022.13 eV (liquid electrolyte) to 1021.65 eV (PC/CTS electrolyte), indicating electronic interactions between zinc species and the organic functional groups, further corroborating interfacial chemical modification. Collectively, the XPS analysis reveals that PC and CTS molecular chains co-adsorb onto the zinc anode surface, constructing a dynamic composite interfacial layer that both blocks water contact and regulates ion deposition behavior.

**Figure 5:**
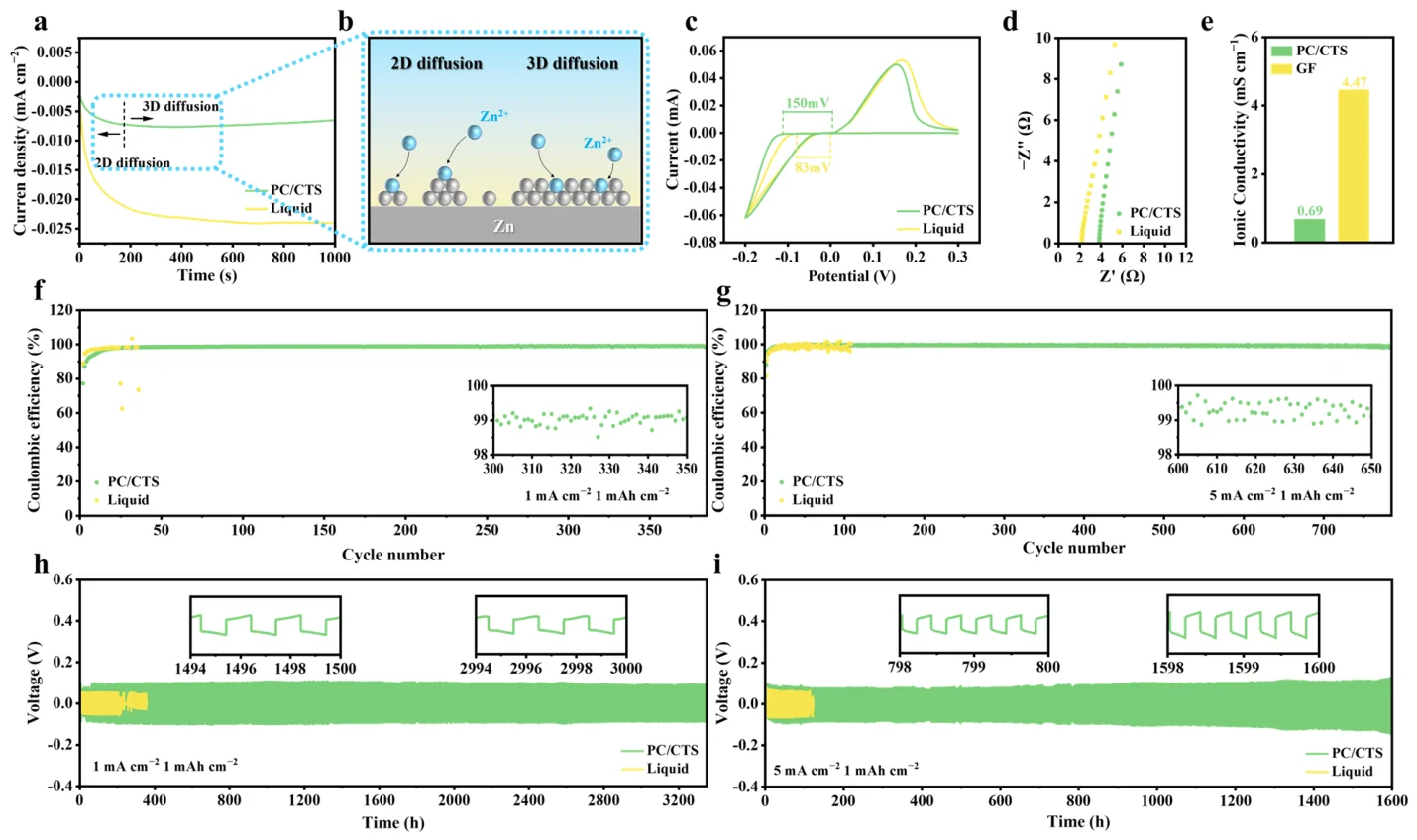
(**a**) CA curves; (**b**) Schematic diagrams of two dimensional and three dimensional diffusion, blue spheres represent Zn^2+^ ions, silver spheres represent metallic Zn atoms; (**c**) CV curves of Zn//Cu half cells assembled with the liquid 2 M ZnSO_4_ electrolyte and the PC/CTS hydrogel electrolyte; (**d**,**e**) Ionic conductivity of the liquid 2 M ZnSO_4_ electrolyte and the PC/CTS hydrogel electrolyte; CE of Zn//Cu half cells assembled with the liquid 2 M ZnSO_4_ electrolyte and the PC/CTS hydrogel electrolyte at (**f**) 1 mA cm^−2^ and 1 mAh cm^−2^ and (**g**) 5 mA cm^−2^ and 1 mAh cm^−2^; (**h**) Cycling performance of Zn//Zn symmetric cells assembled with the liquid 2 M ZnSO_4_ electrolyte and the PC/CTS hydrogel electrolyte at 1 mA cm^−2^ and 1 mAh cm^−2^, and (**i**) at 5 mA cm^−2^ and 1 mAh cm^−2^.

Chronoamperometry (CA) measurements (Figure 5a,b) provide direct evidence of the PC/CTS electrolyte’s ability to homogenize Zn^2+^ flux during deposition. In the liquid electrolyte, the current density continuously increases over 190 s, characteristic of rampant two-dimensional (2D) diffusion driven by the tip effect, ultimately leading to dendrite formation. In contrast, the PC/CTS electrolyte shows a minimal current that rapidly reaches a steady state and remains at a low level, indicating that Zn^2+^ transitions to a three-dimensional (3D) diffusion mode, a hallmark of uniform nucleation and growth [42,43]. Cyclic voltammetry (CV) of Zn//Ti half cells (Figure 5c) corroborates this, showing that the PC/CTS electrolyte maintains a higher zinc nucleation overpotential compared to liquid electrolyte, which promotes smaller nucleation size and more uniform deposition distribution, thereby effectively suppressing dendrite formation. This phenomenon is closely related to the guiding effect of the functional groups on Zn^2+^ transport. Ionic conductivity measurements (Figure 5d,e) show that the PC/CTS electrolyte exhibits slightly lower conductivity than the liquid electrolyte (0.69 vs. 4.47 mS cm^−1^), which is expected for a quasi-solid electrolyte due to the restrictive effect of the polymer network on ion migration, and is sufficient for practical battery operation.

The reversibility of zinc deposition/dissolution was evaluated in Zn//Cu asymmetric batteries. The PC/CTS electrolyte achieves an average CE of 99.07% over 380 cycles at 1 mA cm^−2^ and 1 mAh cm^−2^, with stable voltage plateaus, substantially outperforming the liquid ZnSO_4_ electrolyte, which exhibits fluctuating CE and early failure after approximately 100 cycles (Figure 5f and Figure S10). Even under harsher conditions (5 mA cm^−2^ and 1 mAh cm^−2^), the PC/CTS electrolyte maintains a high average CE of 99.54% over 780 cycles (Figure 5g and Figure S11). The average CE slightly below 99.5% is attributed to the lower ionic conductivity of the quasi-solid hydrogel, minor irreversible capacity associated with the dynamic adsorption layer, and low-rate residual hydrogen evolution and corrosion, all of which are substantially mitigated relative to the liquid system. Long-term cycling tests show that the PC/CTS-based Zn//Zn symmetric cell operates stably for 3350 h at 1 mA cm^−2^ and 1 mAh cm^−2^ (Figure 5h) and for 1600 h at 5 mA cm^−2^ and 1 mAh cm^−2^ (Figure 5i), significantly outperforming the liquid ZnSO_4_ system. The short lifetime of the liquid system (up to approximately 400 h) originates from water-induced side reactions: hydrogen evolution raises the local pH, promoting precipitation of insulating Zn_4_SO_4_(OH)_6_·xH_2_O and non-uniform deposition, which, together with the tip effect, accelerates dendrite growth and eventually causes cell failure. In the PC/CTS hydrogel, restricted water activity, uniform Zn^2+^ transport, and a dynamically adsorbed protective layer suppress these processes. The gradual overpotential increase after approximately 600 h reflects slowly increasing polarization rather than instability, and no failure occurred within the tested window. EIS measurements on Zn//Zn symmetric cells further probed the electrode/electrolyte interface (Figure S13, Table S1). The PC/CTS electrolyte exhibited higher interfacial resistance (Rf = 93.33 ohm) and charge-transfer resistance (Rct = 328 ohm) than the liquid 2 M ZnSO_4_ electrolyte (Rf = 49.67 ohm, Rct = 206.2 ohm), consistent with its lower ionic conductivity and the adsorbed polymer interphase on the zinc surface. Despite this impedance increase, the PC/CTS cell cycled stably for 3350 h at 1 mA cm^−2^ and 1 mAh cm^−2^, among the leading values reported for hydrogel electrolytes (Table S2 [22,44,45,46,47,48,49,50,51,52]). This stability reflects the chemical robustness of the polymer network: the electrostatic complexation and hydrogen-bonding network, together with amide crosslinks formed during drying, remain stable under weakly acidic conditions at room temperature. XPS analysis corroborates this interpretation by showing that the polymer components remain present on the zinc surface after cycling. The anchorage-capture mechanism therefore sustains long-term performance through interfacial protection and homogeneous Zn^2+^ flux, despite the moderate kinetic penalty of the quasi-solid electrolyte.

### 2.4. Full Cell Performance and Environmental Sustainability

**Figure 6:**
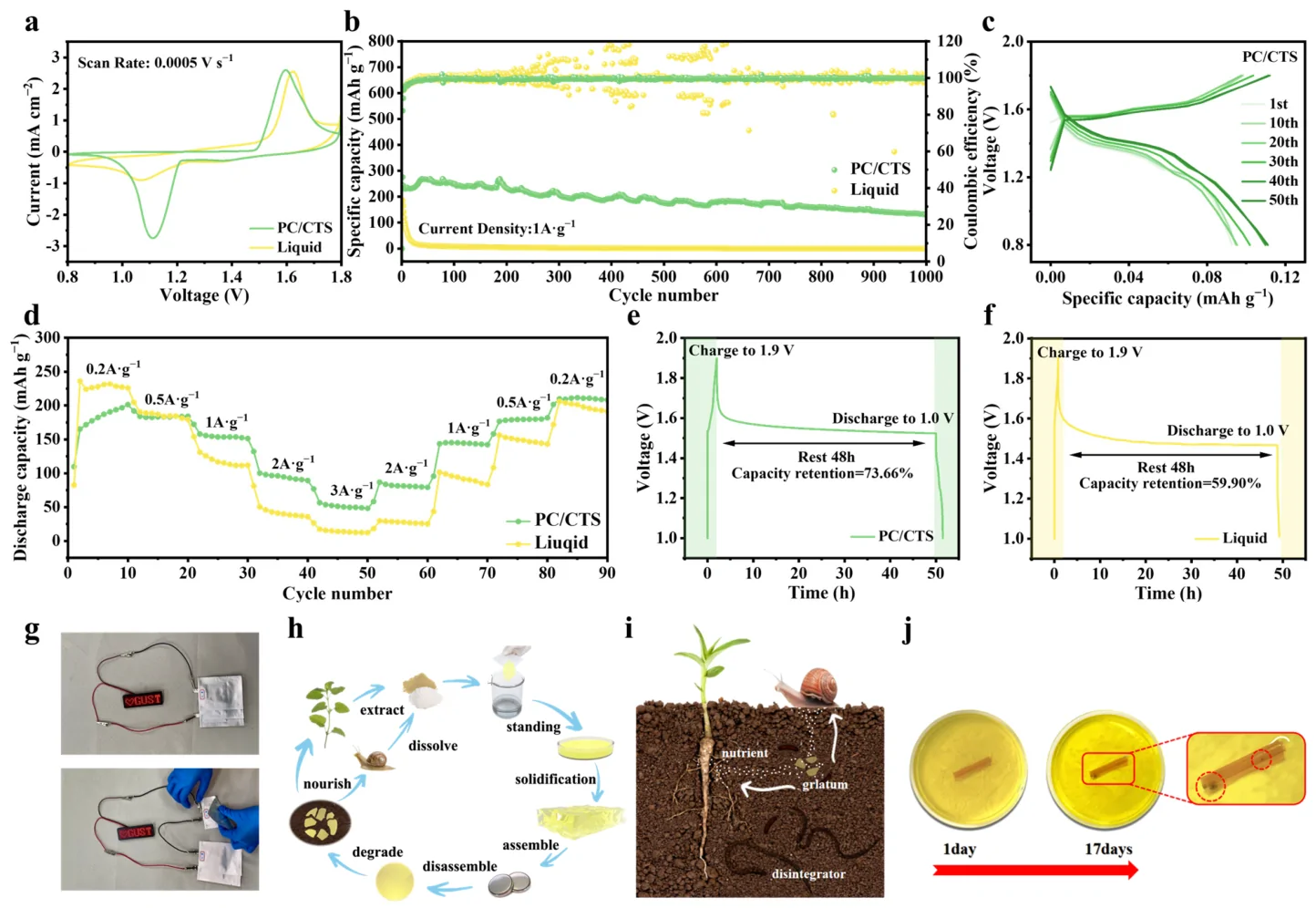
(**a**) CV curves of Zn//MnO_2_ full cells; (**b**) Charge-discharge cycling performance of Zn//MnO_2_ full cells at 1 A g^−1^; (**c**) Capacity-voltage curves of Zn//MnO_2_ full cells assembled with the PC/CTS electrolyte; (**d**) Rate performance of Zn//MnO_2_ full cells using different electrolytes; (**e**) Self-discharge behavior of the PC/CTS electrolyte and (**f**) the liquid electrolyte; (**g**) Photograph of a pouch cell assembled with the PC/CTS electrolyte; (**h**,**i**) Schematic illustrations of the environmentally friendly nature of the PC/CTS electrolyte; (**j**) Photograph showing the biodegradability of the PC/CTS electrolyte; the red circle highlights the region of microbial growth observed after 17 days in Bacillus culture medium.

To validate the practical applicability of the PC/CTS hydrogel electrolyte, we assembled Zn//MnO_2_ full cells using commercial alpha-MnO_2_ as the cathode material without any additional treatment (Figure S14). CV at 0.5 mV s^−1^ (Figure 6a) shows well-defined redox peaks in both electrolyte systems, with the reduction and oxidation peaks for the PC/CTS group located at 1.17 V and 1.33 V, respectively, while those for the liquid electrolyte group are at 1.22 V and 1.35 V. The smaller peak separation in the PC/CTS group indicates lower electrochemical polarization and superior reaction kinetics, attributed to the optimized interfacial environment provided by the hydrogel [8]. Long-term cycling at 1 A g^−1^ (Figure 6b) demonstrates that the PC/CTS-based full cell achieves 63.8% capacity retention after 500 cycles with well-reproducible charge–discharge curves (Figure 6c and Figure S15), significantly outperforming the liquid electrolyte system, which shows rapid capacity fading. Rate performance tests (Figure 6d) show that although the PC/CTS cell exhibits slightly lower capacity during initial cycles due to an activation process, after a brief activation period it demonstrates more stable reversible capacity at all tested current densities, with the advantage particularly pronounced at high current densities, confirming that the gel electrolyte preserves electrode structural stability and reaction reversibility under aggressive cycling conditions [53].

Self-discharge tests (Figure 6e,f) show PC/CTS-based cells retain 73.66% capacity retention after 24 h open-circuit rest, compared to 59.90% for liquid-electrolyte cells, attributable to suppression of cathode dissolution and interfacial side reactions by the hydrogel. Flexible pouch cells with three serially connected units stably power an LED under repeated bending (Figure 6g), confirming mechanical robustness for wearable applications [54].

Biodegradability of the PC/CTS electrolyte was evaluated (Figure 6h–j). A small piece of PC/CTS hydrogel placed in a Bacillus culture medium at room temperature shows distinct bacterial colony formation within 17 days, accompanied by structural decomposition, showing good biodegradability [55]. This environmentally friendly characteristic offers new possibilities for sustainable green energy storage systems. The dual-network strategy can synergistically improve the mechanical properties and cycling stability of hydrogel electrolytes, and the biomass-derived dual network additionally provides sustainability advantages (Table S3 [56,57,58,59,60,61,62,63,64,65]).

## 3. Conclusions

In this work, we designed a fully biomass-derived dual-network hydrogel electrolyte based on PC and CTS, two naturally abundant polysaccharides. The molecular engineering of the polymer network, combining electrostatic crosslinking, hydrogen bonding, and amide bond formation, yields a mechanically robust polymer electrolyte (77.76 MPa tensile strength) with intrinsic dual functionality: the amino groups of CTS anchor to the zinc surface to block parasitic reactions, while the carboxyl groups of PC capture and desolvate Zn^2+^ ions to guide uniform deposition. This anchorage-capture mechanism, arising directly from the polymer molecular design, achieves stable zinc anode cycling for 3350 h and a 63.8% capacity retention in full cells. From a sustainability perspective, the low-cost biomass-derived processing of PC/CTS and the biodegradability of the polysaccharide network provide an environmentally favorable electrolyte component for aqueous zinc batteries, although the zinc anode and MnO_2_ cathode still require conventional recycling routes. It should be noted that the present cycling and reproducibility tests were conducted in laboratory CR2032 coin cells; statistical failure-rate evaluation in commercial cell formats was not performed and remains future work. The dual-network biopolymer strategy demonstrated here provides a generalizable materials platform for sustainable, high-performance polymer electrolytes in aqueous battery systems.

## Data Availability

The data that support the findings of this study are available from the corresponding author upon reasonable request.

## Supplementary Materials

The following supporting information can be downloaded at: https://www.mdpi.com/article/10.3390/polym18161957/s1. Figure S1: Thickness measurements of GF and PC/CTS; Figure S2: Water retention capability; Figure S3: Electrostatic potential maps of PC and CTS molecules; Figure S4: SEM image of the pristine zinc foil before cycling; Figure S5: Surface roughness images of Zn anodes after 50 hours of cycling in (a)liquid electrolyte and (b) PC/CTS electrolyte, and after 100 hours of cycling in (c) liquid electrolyte and (d) PC/CTS electrolyte; Figure S6: C 1s XPS spectra of the zinc anodes cycled in liquid electrolyte at 1 mA cm^−2^ and 1 mAh cm^−2^ for 100 h; Figure S7. O 1s XPS spectra of the zinc anodes cycled in liquid electrolyte at 1 mA cm^−2^ and 1 mAh cm^−2^ for 100 h; Figure S8: S 2p XPS spectra of the zinc anodes cycled in liquid electrolyte at 1 mA cm^−2^ and 1 mAh cm^−2^ for 100 h; Figure S9: Zn 2p XPS spectra of the zinc anodes; Figure S10: Capacity–voltage curves of the Zn//Cu half-cell assembled with the (a) PC/CTS electrolyte and (b) liquid electrolyte at 1 mA cm^−2^ and 1 mAh cm^−2^; Figure S11: Capacity–voltage curves of the Zn//Cu half-cell assembled with the (a) PC/CTS electrolyte and (b) liquid electrolyte at 5 mA cm^−2^ and 1 mAh cm^−2^; Figure S12: Cycling performance of Zn//Zn symmetric cells assembled with PC/CTS electrolyte and liquid ZnSO_4_ electrolyte at 10 mA cm^−2^ and 10 mAh cm^−2^; Figure S13. Nyquist plots of Zn//Zn symmetric cells assembled with the liquid 2 M ZnSO_4_ electrolyte and the PC/CTS hydrogel electrolyte, measured at open-circuit potential before cycling; Figure S14: SEM image of the MnO_2_ cathode; Figure S15: Charge–discharge cycling performance of Zn//MnO_2_ full cells at 1 A g^−1^; Table S1. EIS fitting results of Zn//Zn symmetric cells assembled with the liquid 2 M ZnSO_4_ electrolyte and the PC/CTS hydrogel electrolyte, measured at open-circuit potential before cycling [22,44,45,46,47,48,49,50,51,52]; Table S2. The electrochemical performance comparison between the PC/CTS hydrogel electrolyte and other reported hydrogel electrolytes for ZMBs (Zn//Zn cell); Table S3. The operating mechanisms and advantages and disadvantages of different types of gel electrolytes [56,57,58,59,60,61,62,63,64,65].
