# Supplementary material for: Anchorage-Capture Dual Mechanism in a Biomass-Derived Hydrogel Electrolyte for Dendrite-Free Aqueous Zinc Ion Batteries
Source: Polymers (Basel). 2026 Aug 10;18(16):1957. doi: 10.3390/polym18161957 (PMC13516691; doi:10.3390/polym18161957)
Supplement: Supplementary file 1 [file polymers-18-01957-s001.zip › polymers-4448589-supplementary.pdf]

Supporting information

Anchorage-Capture Dual Mechanism in a Biomass Derived Hydrogel Electrolyte for Dendrite Free Aqueous Zinc Ion Batteries

Experimental Section

Chemicals and materials: Chitosan (deacetylation degree ≥95%, viscosity 100–200 mPa·s, Aladdin, Shanghai, China), pectin (galacturonic acid (dry basis) ≥74.0%, Aladdin), acetic acid (36%, AR grade, Aladdin), manganese(II) sulfate monohydrate ($MnSO_4·H_2O$, AR grade, Aladdin), manganese(IV) oxide (α-MnO_2, purity >98%, Future Material, Shenzhen, China), zinc foil (thickness 100 μm, purity >99.99%, Qingyuan, China), titanium foil (thickness 20 μm, Qingyuan), zinc sulfate heptahydrate ($ZnSO_4·7H_2O$, AR grade, Aladdin), and glass fiber separator (GF-A, Whatman). All chemicals were used as received without further purification. Deionized water (18.2 MΩ·cm) was used throughout all experiments.

Preparation of PC/CTS: The PC/CTS dual-network hydrogel was prepared via a solution-casting method. In a typical procedure, 0.2 g of pectin (PC) powder was dissolved in 40 mL of deionized water under magnetic stirring at 500 rpm for 30 min at room temperature to obtain a homogeneous solution. Subsequently, 0.4 g of chitosan (CTS) powder was added to the PC solution, and the mixture was stirred for 5 min to ensure uniform dispersion. Then, 0.1 mL of 36% acetic acid solution was added dropwise to initiate the electrostatic complexation between the protonated amino groups of CTS and the carboxylate groups of PC. The resulting mixture was stirred vigorously for 6 h at room temperature to ensure complete crosslinking and homogeneity. The obtained chitosan/pectin solution was then transferred into a glass Petri dish (9 cm diameter) and dried in an oven at 45 ºC for 24 h to form a free standing hydrogel membrane. After drying, the membrane was carefully peeled off using tweezers and cut into pieces of the desired size for further use.

Preparation of the electrolytes: A 2 M $ZnSO_4$ aqueous electrolyte was prepared by dissolving 17.32 g of $ZnSO_4·7H_2O$ in 22.44 mL of deionized water under magnetic stirring until complete dissolution. For the full cell electrolyte, 0.0845 g of $MnSO_4·H_2O$ was added to 5 mL of the prepared 2 M $ZnSO_4$ solution as a capacity stabilizer. The PC/CTS hydrogel electrolyte was obtained by immersing the as-prepared PC/CTS membrane in 2 M $ZnSO_4$ electrolyte for 12 h prior to cell assembly, allowing complete absorption of the liquid electrolyte into the polymer network. The conventional liquid 2 M $ZnSO_4$ electrolyte (with a glass fiber separator) and the PC/CTS hydrogel electrolyte were used throughout this study.

Materials characterization: Surface morphology and cross-sectional microstructure were examined using a Tescan Mira scanning electron microscope (SEM, Tescan, Czech Republic) at 5 kV accelerating voltage. X-ray diffraction (XRD) patterns were collected on a Rigaku SmartLab SE diffractometer (Rigaku, Japan) using Cu Kα radiation (λ = 1.5406 Å) at 40 kV and 40 mA, with a scan speed of 5° min^{-1} over 2θ = 5–80°. FTIR spectra were recorded on a Thermo Scientific iN10 spectrometer in ATR mode over 400–4000 cm^{-1} at 4 cm^{-1} resolution. Raman spectra were acquired using a LabRAM HR Evolution (Horiba, France) with 532 nm laser excitation. ^1H NMR spectra were recorded on a Bruker Avance III 400 MHz spectrometer. XPS was performed on a Thermo Scientific K-Alpha spectrometer using monochromatic Al Kα radiation (1486.6 eV) with binding energies calibrated to C 1s (284.8 eV). Mechanical properties were evaluated using a universal testing machine (CMT6503, MTS, China) at a crosshead speed of 5 mm min^{-1}. The pH values of the PC/CTS casting solution before gelation and of the 2 M $ZnSO_4$ electrolyte and the PC/CTS hydrogel after 12 h immersion were measured using a pH meter (PHS-3C) at room temperature.

Calculation method:

The adsorption energy (E_{ads}) is calculated according to the following equation:

$$E_{ads} = E_{total} - E_{Zn} - E_{surface}$$

where E_{total}, E_{Zn}, and $E_{surface}$ represent the total energy of the system, the energy of the isolated Zn, and the energy of the surface, respectively.

The binding energy is calculated according to the following equation:

$$E_b = E_{total} - E_{Zn^{2+}} - E_{species}$$

whereE_{total}, $E_{Zn^{2+}}$, and $E_{species}$ represent the total energy of the system, the energy of the Zn^{2+}, and the energy of the species, respectively.

Electrochemical measurements: All tests were performed using CR2032 coin cells assembled in ambient air. Zn//Zn symmetric cells were constructed by sandwiching the PC/CTS hydrogel electrolyte (or glass fiber separator soaked with 2M $ZnSO_4$ aqueous electrolyte) between two polished zinc foil electrodes (100 μm thick, 10 mm diameter). Zn//Cu half-cells were assembled with copper foil as the working electrode. Zn//MnO_2 full cells were fabricated with commercial α-MnO_2 as cathode active material, mixed with carbon black (Super P) and PVDF binder (7:2:1 weight ratio) in NMP, coated on titanium foil, and dried at 80 °C for 12 h (mass loading: 2-3 mg cm^{-2}). Galvanostatic cycling was conducted on a LAND CT2001A system. CA, LSV, EIS, Tafel, and CV were recorded using a CHI 760E workstation. CV was performed at 0.5 mV s^{-1} (full cells) and 5 mV s^{-1} (Zn//Cu). EIS was conducted from 0.1 Hz to 100 kHz with 5 mV amplitude. Self-discharge was evaluated by charging to 1.8 V, resting for 24 h, then discharging to 0.8 V.

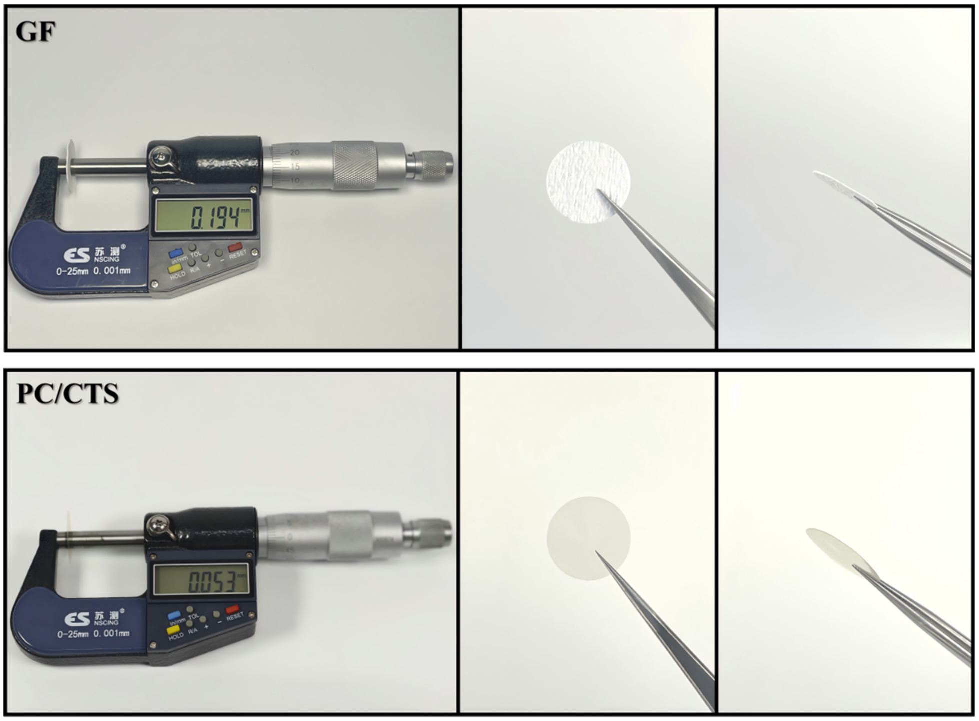

Figure S1. Thickness measurements of GF and PC/CTS.

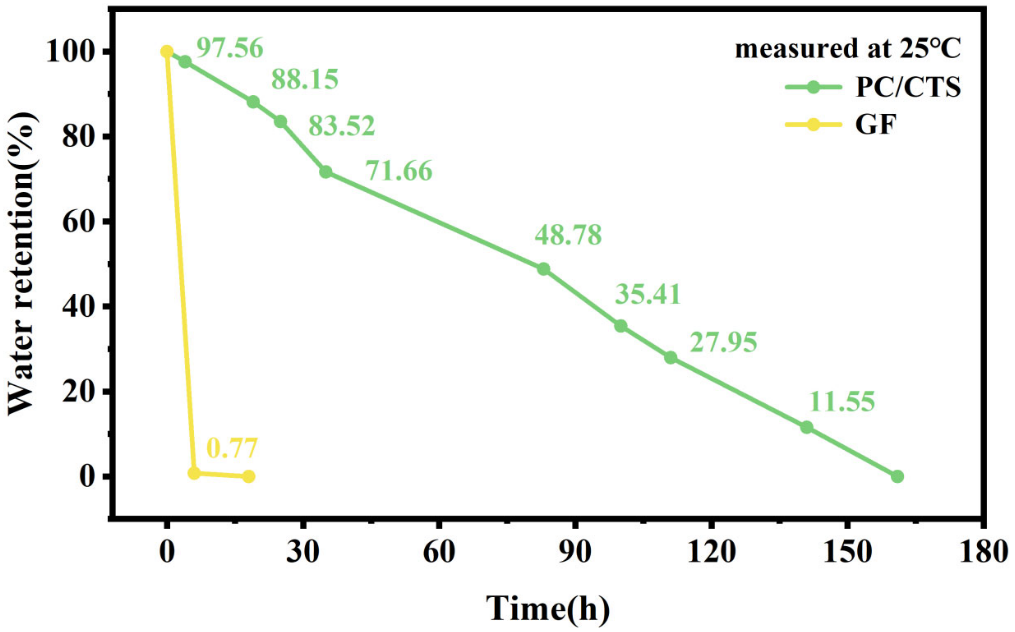

Figure S2. Water retention capability.

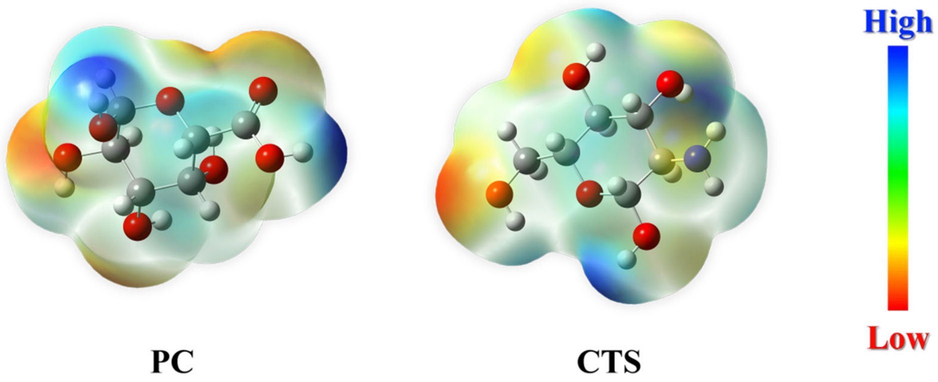

Figure S3. Electrostatic potential maps of PC and CTS molecules;

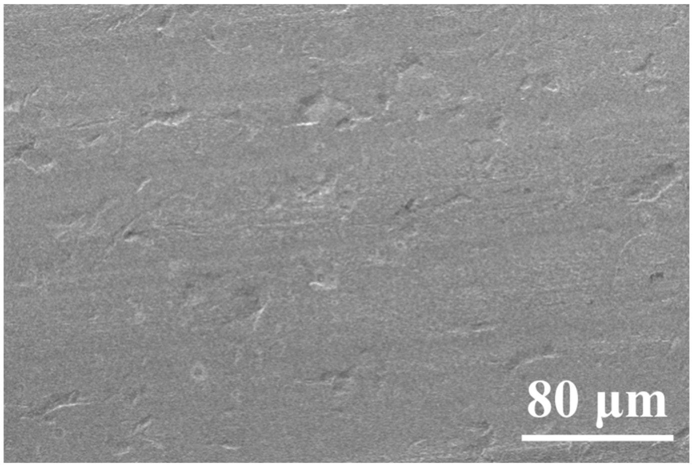

Figure S4. SEM image of the pristine zinc foil before cycling.

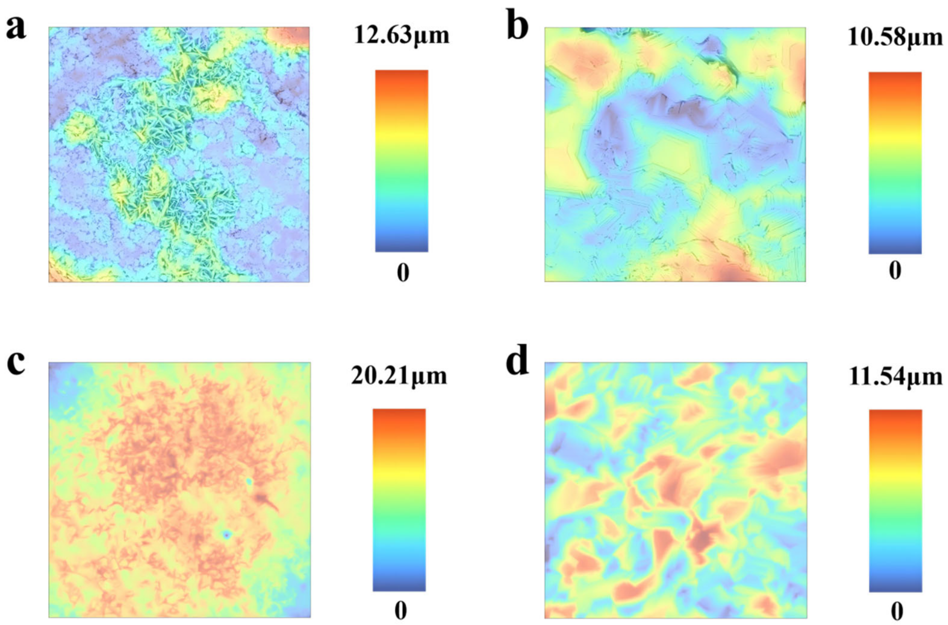

Figure S5. Surface roughness images of Zn anodes after 50 hours of cycling in (a)liquid electrolyte and (b) PC/CTS electrolyte, and after 100 hours of cycling in (c) liquid electrolyte and (d) PC/CTS electrolyte.

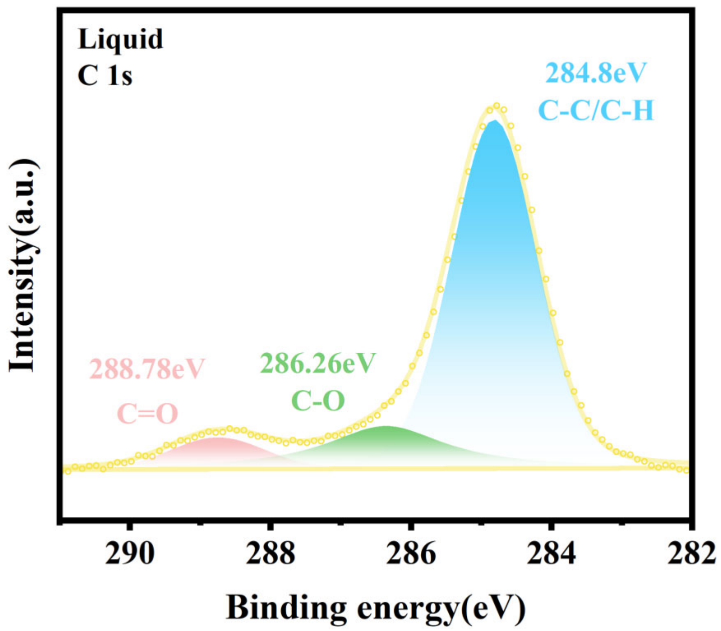

Figure S6. C 1s XPS spectra of the zinc anodes cycled in liquid electrolyte at 1 mA cm^{-2} and 1 mAh cm^{-2} for 100 h.

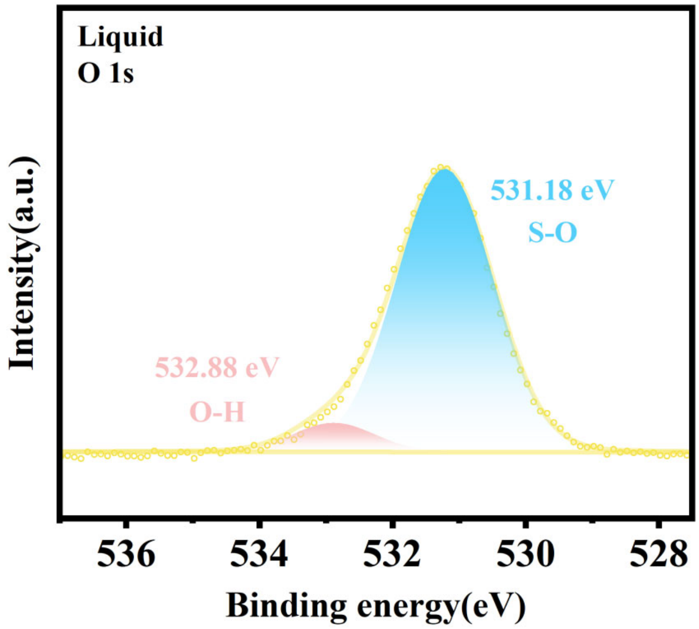

Figure S7. O 1s XPS spectra of the zinc anodes cycled in liquid electrolyte at 1 mA cm^{-2} and 1 mAh cm^{-2} for 100 h.

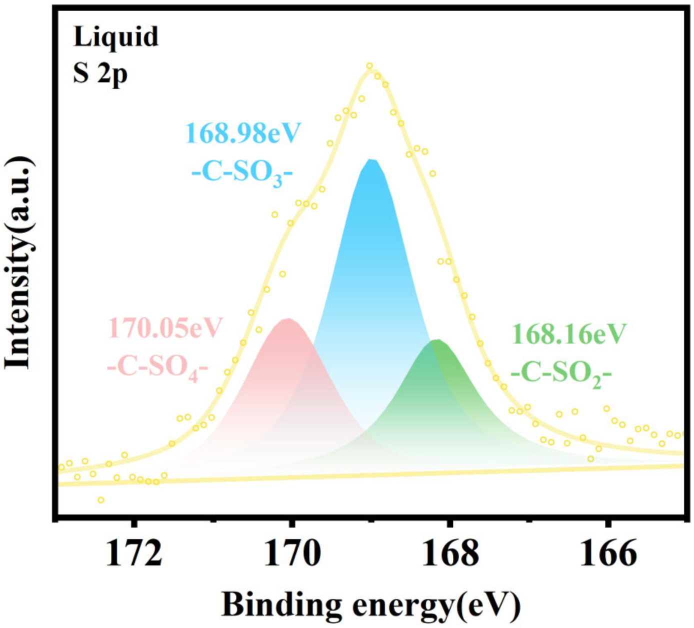

Figure S8. S 2p XPS spectra of the zinc anodes cycled in liquid electrolyte at 1 mA cm^{-2} and 1 mAh cm^{-2} for 100 h.

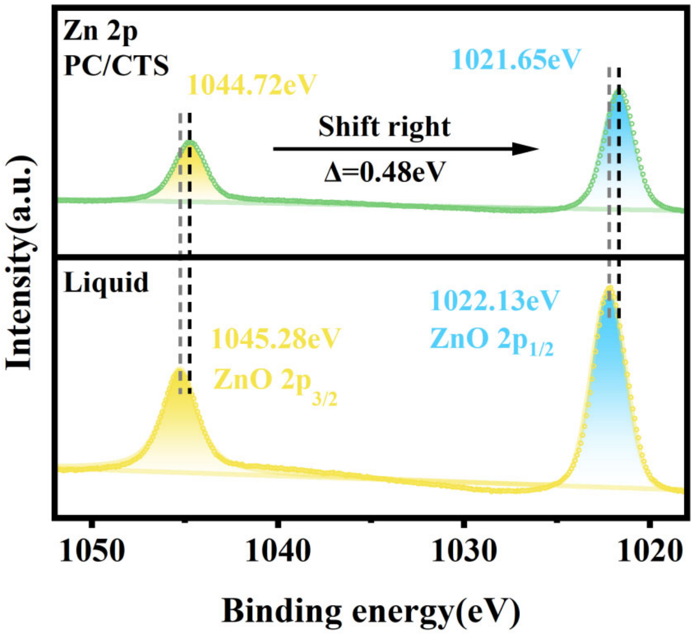

Figure S9. Zn 2p XPS spectra of the zinc anodes cycled at 1 mA cm^{-2} and 1 mAh cm^{-2} for 100 h.

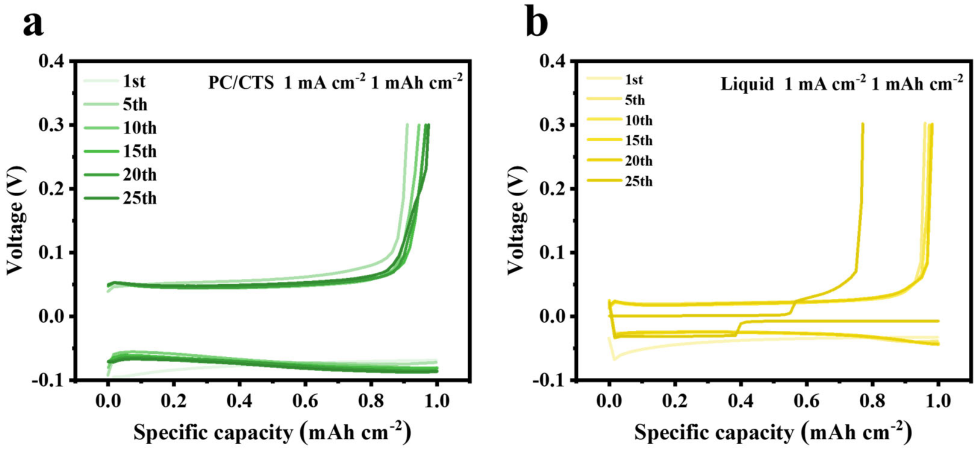

Figure S10. Capacity–voltage curves of the Zn//Cu half-cell assembled with the (a) PC/CTS electrolyte and (b) liquid electrolyte at 1 mA cm^{-2} and 1 mAh cm^{-2}.

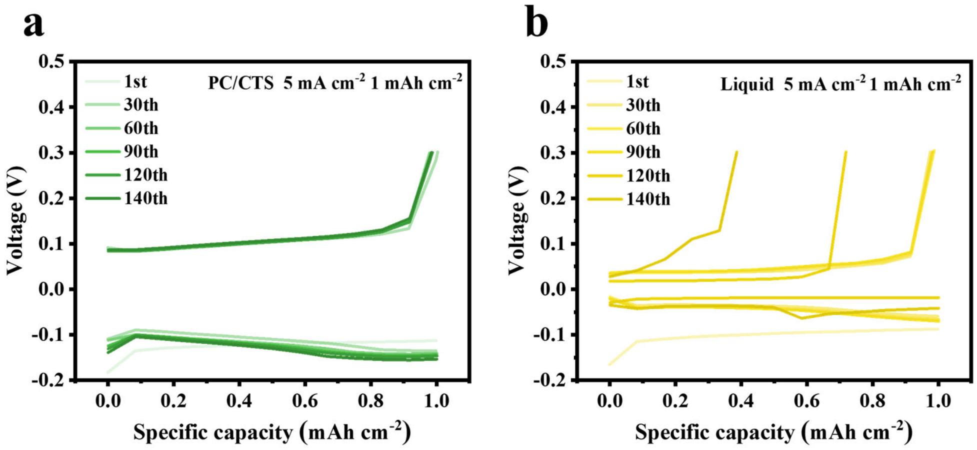

Figure S11. Capacity–voltage curves of the Zn//Cu half-cell assembled with the (a) PC/CTS electrolyte and (b) liquid electrolyte at 5 mA cm^{-2} and 1 mAh cm^{-2}..

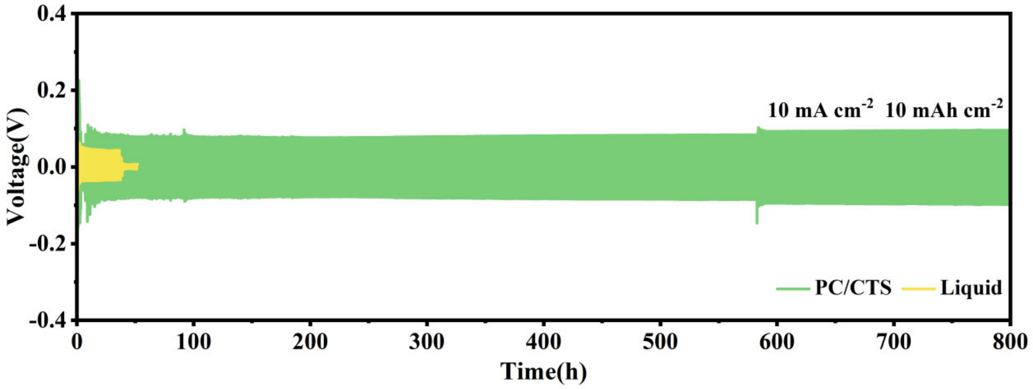

Figure S12. Cycling performance of Zn//Zn symmetric cells assembled with PC/CTS electrolyte and liquid $ZnSO_4$ electrolyte at 10 mA cm^{-2} and 10 mAh cm^{-2}.

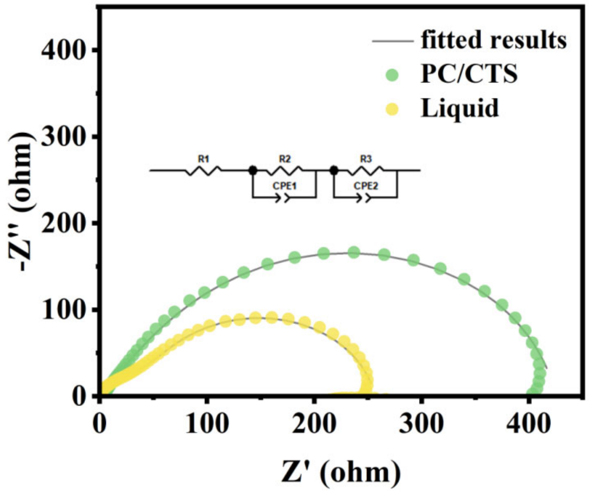

Figure S13. Nyquist plots of Zn//Zn symmetric cells assembled with the liquid 2 M $ZnSO_4$ electrolyte and the PC/CTS hydrogel electrolyte, measured at open-circuit potential before cycling.

Table S1. EIS fitting results of Zn//Zn symmetric cells assembled with the liquid 2 M $ZnSO_4$ electrolyte and the PC/CTS hydrogel electrolyte, measured at open-circuit potential before cycling.

	**Rf (Ω)**	**Rct (Ω)**
PC/CTS	93.33	328
Liquid	49.67	206.2

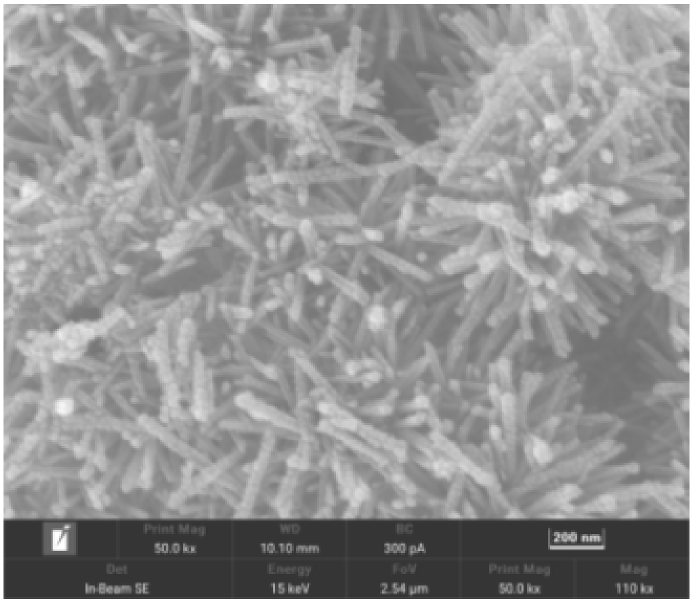

Figure S14. SEM image of the MnO_2 cathode.

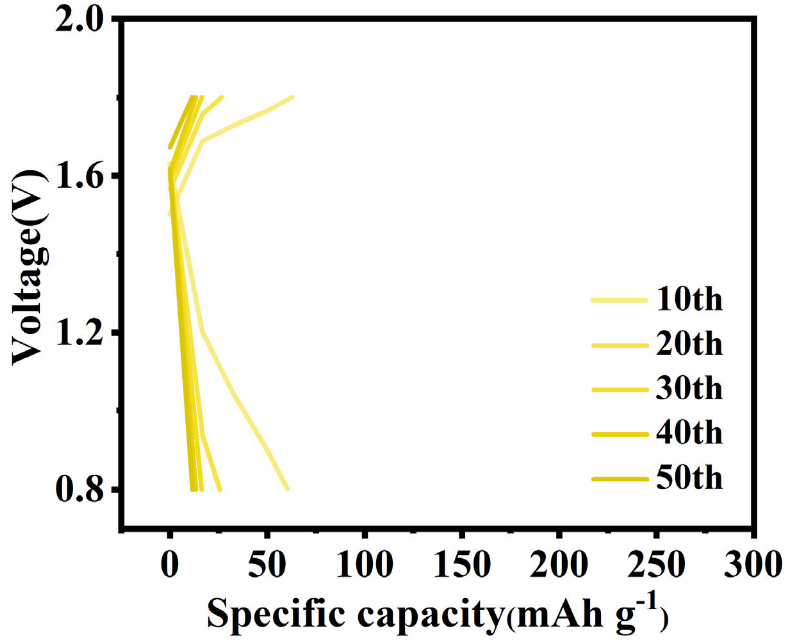

Figure S15. Charge–discharge cycling performance of Zn//MnO_2 full cells at 1 A g^{-1}.

Table S2. The electrochemical performance comparison between the PC/CTS hydrogel electrolyte and other reported hydrogel electrolytes for ZMBs (Zn//Zn cell).

Ref.	Hydrogel / electrolyte	Electrolyte salt	Current density (mA cm^{-2})	Capacity (mAh cm^{-2})	Cycling time (h)
This work	PC/CTS	$ZnSO_4$	1	1	3350
This work	PC/CTS	$ZnSO_4$	5	1	1600
(S1)[22]	DN-based	$ZnSO_4$	1	1	1700
(S2)[44]	DN-based	$ZnSO_4$	1	0.5	1200
(S3)[45]	DN-based	$ZnSO_4$	1	1	2500
(S4)[46]	Amphoteric-based	$Zn(OTF)_2$	1	1	3000
(S5)[47]	Amphoteric-based	$Zn(ClO4)_2$	1	1	1600
(S6)[48]	Cellulose-based	$ZnSO_4$	1	1	2500
(S7)[49]	PAM-based	$ZnSO_4$	1	1	2400
(S8)[50]	PAM-based	$Zn(ClO4)_2$	1	1	>1900
(S9)[51]	PVA-based	$Zn(OTF)_2$	1	0.5	1700
(S10)[52]	PVA-based	$ZnSO_4$	1	1	2200

Table S3. The operating mechanisms and advantages and disadvantages of different types of gel electrolytes.

Ref.	Type	Mechanism	Advantages	Disadvantages
(S11)/ (S12)[56,57]	DN-based	Two interpenetrating networks, one rigid and the other ductile, are crosslinked via physical or chemical bonds. The rigid component bears mechanical load, whereas the ductile counterpart preserves ionic conduction pathways, synergistically endowing the gel with both toughness and high ionic conductivity.	Exhibits outstanding mechanical strength, tear resistance, and stretchability; possesses self-healing capability, rendering it attractive for flexible and wearable devices; achieves a favorable balance between high conductivity and mechanical robustness.	Requires elaborate fabrication procedures and high-cost raw materials; performance becomes unreliable under extreme thermal conditions, limiting its practical applicability.
(S13)/ (S14)[58,59]	Amphoteric-based	The polymer chains bear both cationic and anionic groups, which not only create internal ionic migration pathways but also immobilize water molecules via electrostatic interactions, thereby sustaining adequate hydration within the gel matrix.	Demonstrates functional stability across a broad pH window and retains chemical robustness even under harsh environments; maintains high ionic conductivity over a wide pH range.	Involves multi-step synthesis with stringent processing demands; exhibits a marked decline in conductivity at sub-ambient temperatures, which constrains low-temperature operation.
(S15)/ (S16)[60,61]	Cellulose-based	The intrinsic porosity of the cellulose network facilitates unobstructed ion diffusion, while abundant hydrophilic moieties promote electrolyte uptake and salt dissolution, ensuring steady ionic transport alongside sufficient structural integrity.	Biodegradable and derived from renewable biomass; exhibits good mechanical strength and ready film-forming ability; conductivity can be further enhanced through structural modulation or functionalization.	Performance heavily depends on adequate water content; suffers from degradation under elevated temperatures or extreme pH conditions, which compromises long-term stability.
(S17)/ (S18)[62,63]	PAM-based	The three-dimensional crosslinked polymer network absorbs and retains a large volume of electrolyte, enabling ions to diffuse freely through the	Offers high stretchability and excellent toughness; synthesis is straightforward and cost-effective; exhibits	Experiences severe property deterioration upon water loss; ionic conduction remains poor at low temperatures,

		gel phase and achieve efficient charge transfer between electrodes.	good water solubility, facilitating solution-based processing.	adversely affecting rate capability in cold environments.
(S19)/ (S20)[64,65]	PVA-based	Ion transport is governed by the hydrated polymer network, wherein abundant hydroxyl groups form hydrogen bonds with both water molecules and mobile ions, thereby facilitating ionic migration through the gel matrix.	Cost-effective and non-toxic; exhibits satisfactory electrochemical stability within a reasonable voltage window, making it suitable for conventional battery configurations.	Suffers from insufficient water retention capability; ionic conductivity remains only moderate and often proves inadequate for high-rate or high-energy-density applications.
